# Diagonal earlobe crease and coronary artery disease in a Chinese population

**DOI:** 10.1186/1471-2261-14-43

**Published:** 2014-04-04

**Authors:** Xing-li Wu, Ding-you Yang, Yu-sheng Zhao, Wen-hui Chai, Ming-lei Jin

**Affiliations:** 1 Institute of Geriatric Cardiology, Chinese PLA General Hospital, Beijing, China; 2 Department of Traditional Chinese Medicine, the First Affiliate Hospital of Chinese PLA General Hospital, Beijing, China; 3 Department of Geriatrics, the Changji People’s Hospital, Urumqi, China; 4 Department of Geriatrics, Chengde City People’s Hospital, Hebei, China

**Keywords:** Diagonal earlobe crease, Coronary artery disease, Chinese ethnics, Coronary artery angiography, Han Chinese

## Abstract

**Background:**

Many reports have claimed associations between diagonal earlobe crease (DELC) and coronary artery disease (CAD), but data in Chinese populations are limited.

**Methods:**

This cohort study investigated 449 consecutive Chinese, 250 cases with CAD and 199 without CAD, who were certified by coronary artery angiography in our center. Characteristic differences and the relation of DELC to CAD were assessed by Chi-square and t tests. The multivariate regression was performed to adjust for confounders and ROCs mode were used to detect its predicting performance for CAD.

**Results:**

The prevalence of DELC was 46.2% in those without CAD and 75.2% in those with CAD (P < .001). Subjects with DELC had more stenostic vessels and higher prevalence of both any and significant coronary artery stenosis than those without DELC (P < .001). The sensitivity, specificity and positive and negative predictive values for DELC to diagnose CAD in the whole population were 0.752, 0.538, 0.671 and 0.633. The higher sensitivity and positive predictive values (ppv) were found in male, the lowest sensitivity and the highest ppv in the <45 years old group, and the lowest specificity and ppv in the >75 years old group. After adjusting for other variables including age, gender and traditional risk factors, DELC remained a positive predictor for CAD (OR, 3.408; 95% CI 2.235-5.196; P < 0.001), but not for hypertension, diabetes mellitus, hypercholesterolemia and hypertriglyceridemia. ROC analysis showed the area under the curve was 0.645 (95% CI 0.593-0.697, p < 0.001).

**Conclusions:**

The study showed a significant association between DELC and CAD independent of established risk factors in Chinese.

## Background

Coronary artery disease (CAD) is rapidly increasing in prevalence across the world and particularly in China. With more than 1.3 billion people in China now the morbidity and mortality of CAD is keeping increasing during the past 30 years. It is urgently needed to ascertain a group of simple and reliable predictors of atherosclerosis for the identification of the persons at risk of CAD in the earlier stages in order to lower the burden [1].

The majority of studies have shown a significant association between CAD and the presence of diagonal earlobe creases (DELC) since Frank’s first report in 1973 [2-17]. Regarding myocardial infarction, Lichstein et al. [3] reported in 1974 that DELC (unilateral or bilateral) was significantly more common (47%) in patients with myocardial infarction than those without the disease (30%). Kaukola reported that 69%~72% of patients with an acute myocardial infarction or coronary artery atherosclerosis had a DELC, while in the like-aged control group 21%~24% of them had DELC [4,17]. Studies from Denmark found that the prevalence rate (46.8%) of DELC in those with an acute myocardial infarction was significantly greater than in control group (31.6%) [5].

When patients with CAD as determined by coronary artery angiography (CAG) were taken into consideration, a Turkish study of 415 cases shown a highly significant statistically greater prevalence of DELC (51.4%) in those patients with a positive angiogram (as defined as >70% stenosis of the luminal diameter) than in those whose angiogram was normal (15.1%) [6]. In a Japanese study, DELC was present in 26.1% with stenosis (as defined as >50% stenosis), but in only 3.7% without stenosis [7]. A most recently study in US found that the sensitivity, specificity, and positive and negative predictive values for DELC in detecting CAD as determined by coronary computed tomography angiography were 78%, 43%, 77%, and 45% [8]. The predictive significance of DELC has also been shown in one prospective study consisting of 108 of patients who had at least one DELC but free of CAD. After followed for an 8–10 year period, cardiac event rates for those with DELC were higher (10.4 events per 100 patient-years) than for those without DELC (1.4 events per 100 patient-years) [9]. In a Denmark research consisting of 14,223 healthy persons disclosed that those with the DELC had a 1.4-fold increased risk of developing an AMI after 6.5 years follow-up [5]. A case–control study included 842 men with myocardial infarction and 712 men with non-cardiac diagnoses showed that the relative risk of myocardial infarction for the presence of DELC was 1.37 (95% CI 1.25-1.5 [10].

Postmortem studies also showed a significant positive correlation between the presence of DELC and CAD [11]. In a UK study, the relative risk of a male with DELC having severe coronary artery atherosclerosis defined as 75% stenosis was 1.64~3.65. The sensitivity of bilateral DELC for detecting severe CAD was 62.1% for men and 69.2% for women. The specificity was 65.9% in men and 78.0% in women [12]. A similar British forensic necropsy study demonstrated that those with DELC (unilateral or bilateral) had a risk of cardiovascular cause of death of 1.55~1.74 times higher than those without a crease [13]. A study of 520 individuals in Swedish showed that DELC was strongly predictive of the presence of CAD and was strongly associated with sudden cardiac death in men, but not women [14].

Various prevalence of DELC in different ethnic groups has also been noted [15]. In a US study using police arrest photographs (“Mug shots”), the prevalence of DELC were as follows: Hawaiian-Samoans 0% (0/12), Chinese 21% (6/29), Blacks 37.9% (44/116), Latin-Americans 47.5% (19/40) and Caucasians 50.8% (62/122) [16].

Despite the above supporting results from different studies, the correlation of DELC and CAD was not demonstrated in several series [18,19]. Koracevic and Atanaskovic [19] reported that irrespective of whether patients had unilateral or bilateral DELC or if the crease was deep or superficial (< 1 mm), there was no significant correlation between DELC and clinical CAD (not even a trend toward significance). In a study including 125 consecutive patients in Ireland showed that for the group of patients as a whole, DELC was not significantly related to the presence of CAD [20]. A study of 261 consecutively male patients in US concluded that there was no difference between DELC and CAD [21]. Kuon et al. [22] who evaluated 670 consecutive patients in German reported that 55.9% of patients with DELC and 55% of patients without DELC had CAD as defined by one or more coronary artery vessels having > 70% stenosis (p > 0.05), nearly the same ratio in both groups.

The limitation of the past studies included a relative small sample, without a control population, only patients with myocardial infarction were selected, the examinee’s position to be observed was not consistent, the diagnostic criteria for CAD was not certified by gold-standard as CAG. Most importantly, the criteria for a typical DELC (including unilateral or bilateral, the number, length, depth) were not unanimous, even not mentioned [2-22]. Lastly, few people in Han Chinese ethnic group were involved in the studied population, even though it has been argued that the prevalence of DELC and the relationship between DELC and CAD may be not the same in different human races and nations [7,23,24].

The present study is aimed to find out the association of DELC with CAD diagnosed by CAG, and to determine whether the identification of DELC could be regarded as a simple, practical and economical marker to identify CAD in an earlier time, as reported in other countries and populations.

## Methods

### Patients and criteria

The present study was designed as a cohort study. Subjects were prospectively recruited from consecutive in-hospital cases that underwent CAG in our hospital from Jan. 2012 to Sep. 2012. A total of 449 subjects including 199 persons without CAD and 250 cases of CAD were enrolled in the present study. Exclusion criteria included those who have an earring or piercing. The data of traditional risk factors (age, gender, hypertension, hypercholesterolemia, diabetes mellitus, smoking and alcohol drinking) and DELC presence were assessed by appropriate questionnaires. Written informed consent was obtained from all the participants and the study was approved by China PLA General Hospital medical ethic committee.

CAD was defined by diameter stenosis of >50% in one or more of the epicardial arteries (left main coronary artery, left anterior descending coronary artery, left circumflex coronary artery, and right coronary artery) revealed by CAG. The extent of CAD was evaluated by analyzing the number of ≥2 diseased vessels and the number of vessels with any stenosis (1%~100%) [8]. For CAD severity, we evaluated the presence of significant CAD (>50% stenosis) and any CAD(1%~100%) [8]. Hypertension was defined as systolic blood pressure (SBP) ≥140 mmHg and/or diastolic blood pressure (DBP) ≥90 mmHg. Diabetes mellitus was denoted as fasting blood sugar ≥126 mg/dL. Smoking and alcohol drinking were defined as “current smokers and drinkers”. Hypercholesterolemia was defined as total cholesterol ≥5.72 mmol/L and hypertriglyceridemia was defined as triglyceride >1.7 mmol/L.

The ear lobes were assessed with the patients in sitting position using a modified evaluation sheet designed by Isha Shrestha [25]. The typical DELC was recorded as a deep diagonal crease (>1 mm) extending obliquely from the tragus towards the outer border of the ear lobe, covering at least two-thirds of the ear lobe length. Patients were examined for DELC by 2 trained observers who were blinded to patients’ diagnosis and have consensus on the presence of DELC. The extent of coronary artery stenosis was calculated using calibrating software by 2 expert readers.

### Statistical analysis

Data were expressed as mean ± standard deviation. Comparison of the categorical or numeric variables between groups was carried out using a chi-squared test or student *t* test separately. The sensitivity, specificity and predictive values of DELC in two gender and four age groups (< 45, 45–59, 60–75 and > 75 years old) were computed from relevant four-fold tables. The multivariate regression model was used to rule out possible mutual association of traditional risk factors (including age, sex, cigarette smoking, alcohol drinking, hypertension, hypercholesterolemia, hypertriglyceridemia and diabetes mellitus) with both CAD and the factors mentioned above. The receiver operator characteristic curves (ROCs) were used to detect indirectly the diagnostic effect of DELC for CAD. A *P* value of < 0.05 was regarded as being statistically significant. All statistical analysis was conducted using SPSS (Statistical Package for Social Sciences, 19.0) for Windows.

## Results

The characteristics of 449 patients (61.9% were male), 280 with DELC and 169 without DELC are shown in Table 1. Patients with DELC were more in male, significantly older, had a higher prevalence of smoking and alcohol drinking. There was no difference in prevalence of other risk factors in terms of DELC (Table 1). The prevalence of DELC was 46.2% in those without CAD and 75.2% with CAD (Table 2).

**Table 1:** Basic clinical characteristics of the subjects with and without DELC

**Variable**	**All**	**With DELC**	** Without DELC**	**x**^**2 **^**or *t***	***P*** *
	**n = 449**	**(n = 280, 62.4%)**	**(n = 169, 37.6%)**		
Male	278 (61.9%)	192 (68.6%)	86 (50.9%)	13.877	0.003
Age (years)	63.29 ± 11.952	65.03 ± 10.65	59.65 ± 12.90	−4.055^**#**^	0.000
Hypertension	315 (70.2)	201 (71.8%)	114 (67.5%)	0.938	0.333
Hypercholesteremia	142 (31.6)	86 (30.7%)	56 (33.1%)	0.285	0.593
Hypertriglycerimia	159 (35.4%)	98 (35.0%)	61 (36.1%)	0.055	0.814
Diabetes mellitus	117 (26.1%)	77 (27.05%)	40 (23.7%)	0.810	0.368
Smoking	155 (34.5%)	109 (38.9%)	46 (27.2%)	6.507	0.011
Alcohol drinking	120 (26.7%)	87 (31.1%)	33 (19.5%)	7.393	0.007

All numbers represent percent prevalence unless otherwise specified. *p Value between subgroups with and without DELC. ^#^analyzed using Student *t* test.

**Table 2:** Clinical comparison of subjects with and without CAD

**Variable**	**All**	**With CAD**	**Without CAD**	**x**^**2 **^**or *t***	***P*** *
	**n = 449**	**(n = 250, 55.7%)**	**(n = 199, 44.3%)**		
Male	278(61.9%)	175(70.1%)	103(51.8%)	15.642	0.000
Age (years)	63.29 ± 11.952	63.74 ± 11.316	62.71 ± 12.71	2.068^**#**^	0.362
Hypertension	315(70.2%)	178(71.2%)	137(68.8%)	0.293	0.588
Hypercholesteremia	142(31.6%)	94(37.6%)	48(24.1%)	9.450	0.002
Hypertriglycerimia	159(35.4%)	100(30.0%)	59(27.6%)	5.232	0.022
Diabetes mellitus	117(26.1%)	81(32.4%)	36(18.1%)	12.065	0.001
Smoking	155(34.5%)	97(38.8%)	58(29.1%)	4.604	0.032
Alcohol drinking	120(26.7%)	75(30.0%)	45(22.6%)	3.116	0.078
DELC	280(62.4%)	188(75.2%	92(46.2%)	39.914	0.000

All numbers represent percent prevalence unless otherwise specified. *p Value between subgroups with and without CAD. ^#^analyzed using Student *t* test.

As for CHD extent and severity, the sample showed that subjects with DELC had more stenostic vessels and higher prevalence of both any and significant coronary artery stenosis than those without DELC (Table 3).

**Table 3:** Extent and severity of CAD in subjects with and without DELC

**CAD measurement**	**With DELC**	**Without DELC**	**x**^**2 **^**or *t***	***P*** *
	**(n = 280, 62.4%)**	**(n = 169, 37.6%)**		
Any CAD	190(67.9%)	81(47.9%)	17.402	0.000
Significant CAD	188(67.1%)	62(36.7%)	39.914	0.000
≥2 diseased vessels	185(66.1%)	62(36.7%)	37.059	0.000
Number of diseased vessels	1.55 + 1.311	0.54 ± 0.852	−8.869^#^	0.000

All numbers represent percent prevalence unless otherwise specified. *p Value between subgroups with and without DELC. ^#^analyzed using Student *t* test.

The sensitivity for the whole population was 75.2%, the specificity 53.8%, the positive predictive value 67.1% and the negative predictive value 63.3%. For gender difference, higher sensitivity and positive predictive values were found in male that that in female, while higher specificity and negative predictive values were in female than that in male. For age difference, the lowest sensitivity, 0.60, and the highest ppv, 0.818, were found in the < 45 years old group. On the contrary, the > 75 years old group had the lowest specificity, 0.379, and a ppv of 0.641. The complete data are seen in Table 4.

**Table 4:** The sensitivity, specificity, and positive and negative predictive values (ppv and npv) for DELC to predict CAD in gender and age groups

**Group**	**Sensitivity**	**Specificity**	**ppv**	**npv**
All	0.752	0.538	0.671	0.633
Male	0.789	0.476	0.719	0.570
Female	0.667	0.604	0.568	0.699
<45 y	0.600	0.875	0.818	0.700
45–59 y	0.676	0.600	0.667	0.610
60–75 y	0.776	0.500	0.677	0.623
> 75 y	0.854	0.379	0.641	0.667

**Figure 1:**
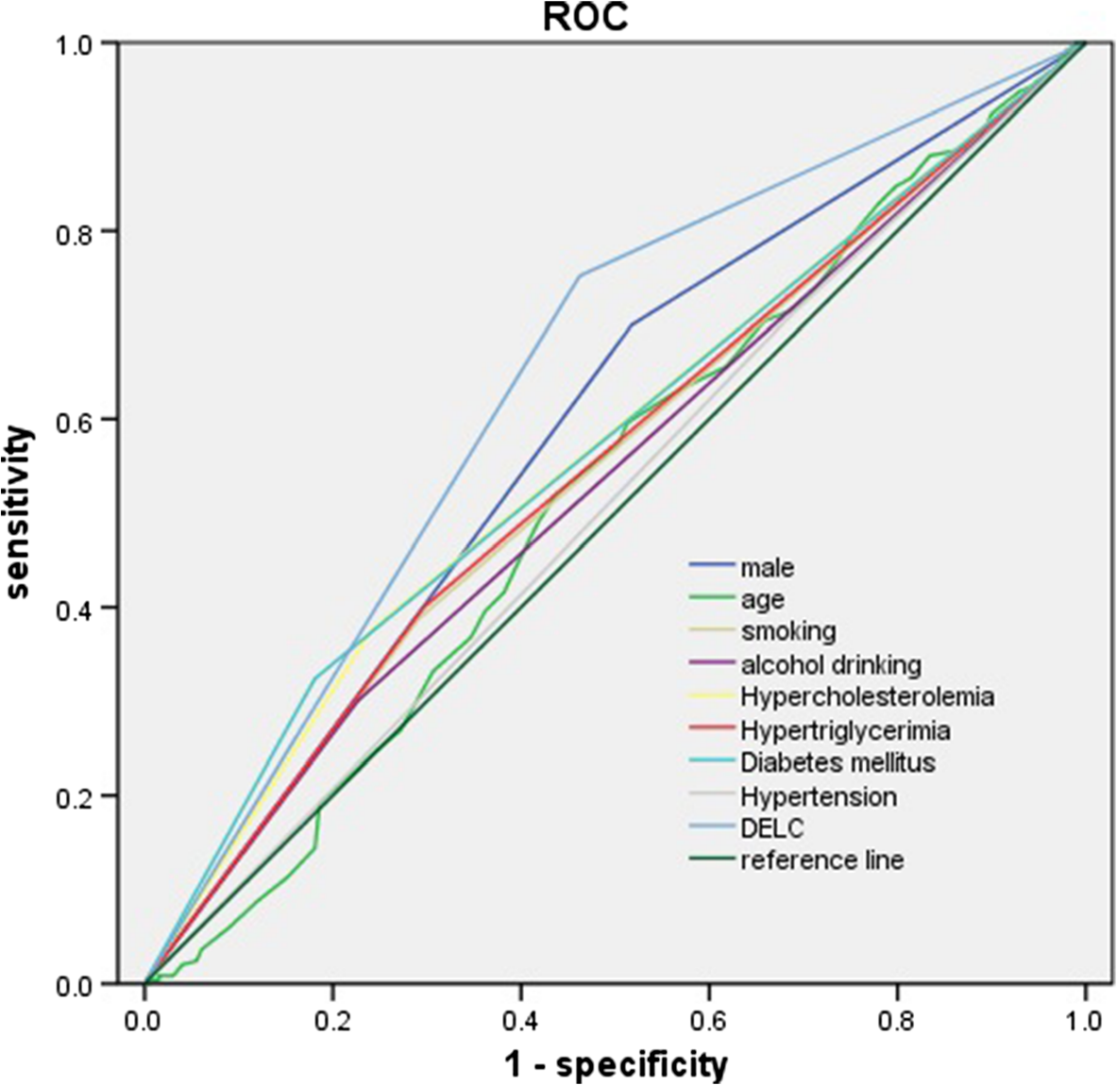
**The separate discriminating effect of all 9 traditional coronary risk factors including DELC for CAD.** The ROC curves were drawn based on the multivariate regression equation which included male gender, age, hypertension, diabetes mellitus, hypercholesterolemia, Hypertriglycerimia, Smoking, Alcohol drinking and DELC. It was used to detect indirectly the discriminating effect of DELC for CAD.

**Figure 2:**
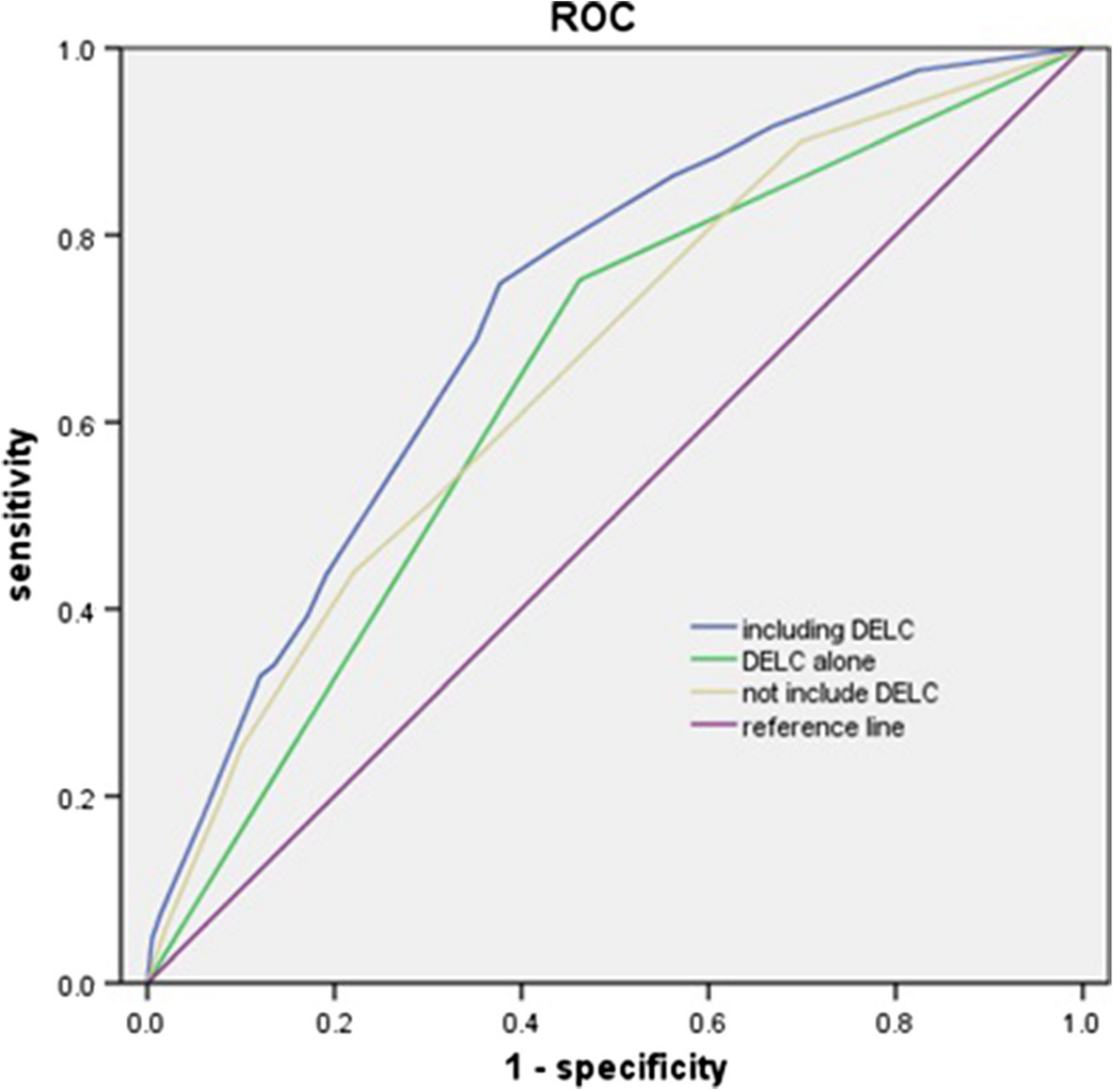
**The discriminating effect of traditional coronary risk factors adding DELC or not for CAD.** The ROC curves were drawn based on the multivariate regression equation which included male gender, diabetes mellitus, hypercholesterolemia and DELC added or not. It was used to detect indirectly the discriminating effect of DELC for CAD.

The ROCs analysis showed that area under the curve for DELC to discriminate CAD was 0.645, for the combined traditional risk factors was 0.660, and 0.722 when adding DELC (Figures 1, 2, Tables 5 and 6).

**Table 5:** The univariate regression analysis and AUCs of risk factors for CAD

**Factors**	**OR**	**95% CI**	***P***	**AUC**	**CI**	***P***
Male	2.206	1.321-3.683	0.002	0.591	0.538-0.644	0.001
Age (years)	0.999	0.981-1.018	0.958	0.523	0.470-0.579	0.369
Smoking	0.927	0.514-1.674	0.803	0.548	0.495-0.602	0.079
Alcohol drinking	0.871	0.474-1.602	0.657	0.537	0.483-0.590	0.179
Hypercholesteremia	2.540	1.173-5.502	0.018	0.567	0.514-0.620	0.014
Hypertriglycerimia	0.835	0.400-1.743	0.631	0.552	0.498-0.605	0.059
Diabetes mellitus	2.413	1.486-3.919	0.000	0.572	0.519-0.624	0.009
Hypertension	1.042	0.656-1.655	0.861	0.512	0.458-0.566	0.668
DELC	3.452	2.234-5.336	2.452	0.645	0.593-0.697	0.000

**Table 6:** The AUCs of risk factors including DELC or not for CAD

**Factors**	**AUC**	**95% CI**	***P***
9 factors including DELC	0.722	0.674-0.769	0.000
8 factors not including DELC	0.660	0.710-0.711	0.000
DELC alone	0.645	0.593-0.697	0.000

All the 9 factors are the same as in Table 2.

The multivariate regression analysis showed that DELC, male gender, diabetes mellitus and hypercholesterolemia were all independent risk factors for CAD. DELC was not a predictor for diabetes mellitus, hypertension, hypercholesterolemia and hypertriglyceridemia (Table 7).

**Table 7:** The multivariate regression analysis of risk factors for CAD

**Factors**	**OR**	**95% CI**	***P***
Diabetes mellitus	2.423	1.495-3.928	0.000
Male gender	2.012	1.320-3.056	0.001
Hypercholesterolemia	2.151	1.373-3.368	0.001
DELC	3.408	2.235-5.196	0.000

## Discussion

Since the first report of DELC by Frank in 1973, various studies have found varying degrees of association between DELC and CAD as reviewed by Friedlande [26], which may reflect differences in age groups, extent of CAD, ethnic distribution, study designs (including clinical or postmortem) and criteria of diagnosing CAD (based on the presence of chest pain, risk factors, electrocardiographic abnormalities at rest, or CAG), not clearly defined DELC (prone or sitting position, depth, length, numbers, unilateral or bilateral). When it comes to other diseases accompanying DELC, a few results have been reported by different authors. They may include the thickened carotid IMT in a population sample of middle-aged adults without known atherosclerotic disease, more cardiac event morbidity and mortality, higher preoperative risk, increased arterial stiffness, metabolic syndrome, and the development of a first acute myocardial infarction (AMI) as followed for a 6.5 year interval after controlling for age and sex [4,5,7,22-24,27,28].

There are about 14 articles written in Chinese language on this topic, but the subjects involved were neither diagnosed by gold criteria for CAD nor unanimous definition for DELC. Our study population is comprised solely of Han Chinese person. Coronary artery stenosis was diagnosed by using CAG and the DELC was strictly defined. The main finding of the present study is that DELC was significantly associated with CAD but not diabetes mellitus, hypertension, hypercholesterolemia and hypertriglyceridemia. The study showed also that subjects with DELC had more stenostic vessels and higher prevalence of both any and significant coronary artery stenosis than those without DELC. To the best of our knowledge, this is the first report regarding the association between DELC and CAG-certified CAD in China mainland.

In the present study, patients with DELC were more in male, significantly older and had a higher prevalence of alcohol drinking. Although hypercholesterolemia and diabetes mellitus were certified unsurprisingly to be the strong risk factors for CAD in this study, we found no difference in the prevalence of them in terms of DELC.

When hypertension was taken into consideration, our study didn’t support the positive correlation result obtained by others [7,29,30]. However, it is in consistent with Jords and Elliotts who failed to find associations between cardiovascular risk factors including hypertension and earlobe in 1000 patients with CAD and 67 pairs of persons with or without DELC (matched by age and sex) [28,31]. As for hypercholesterolemia and hypertriglyceridemia were concerned, a Japanese study [7] suggested a strong correlation with CAD (in the subgroup not certified by CAG). But the present data detected no relation, which went along with Jords and others [4,27,31], and also the result of the same Japanese study [7] (only when CAD being defined by CAG were included). Being not in line with other studies [7,26-28], we found DELC was not related with diabetes mellitus as reported by Kaukola and others samples involving both CAD and non CAD population [4,28,32].

The association between DELC and smoking was reported by Toyosaki and Doering [7,33], but was questioned by other researchers [4,28,31,34] and the present data. In this study, a higher prevalence of alcohol drinking in patients with DELC was detected, but it’s not an independent predictor for DELC or CAD. This is not in accordance with Petrakis who reported a negative association between DELC and alcohol use in white women under 59 years of age and speculated that alcohol may maintain the patency of the end-arteries supplying the myocardium and the earlobe [34].

In terms of gender, our results showed that DELC was predominantly distributed in male sex, this didn’t echoed the clinical and postmortem finding which reported almost equally distribution in both sexes [7,8,13]. On par with studies previously documented [7,14,20,23,31] we also found an increasing trend of DELC prevalence with increasing age, but age was not a positive predictor for CAD and the relationship of DELC with CAD was independent of age and sex. Regarding race, the attainable results were confounding. Postmortem study reported the prevalence of DELC up to 55% to 65% in Sweden and US [11,14], while it was 55% in south Indian population, 5% in native Japanese and 31.1% in healthy male Malaysian [23]. In a USA study [16] using police arrests photographs, the rates of DELC were from 0% (0/12) in Hawaiian-Samoans to 50.8% (62/122) in Caucasians. In our study, the prevalence of DELC was 51.6% in those without CAD and 68.9% in those with CAD, which was almost the same as in US and much higher than in Japanese [7,11]. The higher ratio of DELC may partly attribute to much elder patients involved in our study (63.3 years vs. 51.9 years [11].

Targeting the diagnostic performance for DELC to CAD, the present work showed a similar specificity and negative predictive value, but a lower sensitivity and positive predictive value when compared with recently accomplished observation by Shmilovich [8]. The discrepancy may come from different diagnostic methods and severity of CAD in the two samples. In Shmilovich’s study CAD was certified by computed tomography angiography and any CAD, which means the presence of any coronary artery plaque instead of significant stenosis (>50%), was analyzed. In fact, the positive predictive value to any CAD (including all stenosis from 1% to 100%) in our data increased to 67% (data not shown). The bigger AUC on ROC analysis when DELC was added to the traditional risk factors also reflect an additional discriminating value of DELC in this series. For gender difference, our data showed higher sensitivity and positive predictive values in male than in female, which was not supported by Edston [14]. But the age impact was almost the same as Edston [14], in both of them the lowest sensitivity and the highest ppv were found in the < 45 years old group, while the >75 years old group had the lowest specificity and ppv. The higher ppv in male and in the group below 45 years of age appear to be most useful for screening of younger men, while the higher npv in the group above 75 years of age seems to be helpful to exclude the elderly from the risk of having CAD.

Though most of the above evidence supported the correlation between DELC and CAD, the underlying pathophysiological mechanisms is still unclear [26-38]. The suggested items might include degeneration of elastin and unbalanced ratio of collagen to elastin. As these traits which reflects microvascular disease were similarly seen in biopsy specimens taken from the earlobes and the coronary bed. The primary role of ageing was mostly postulated, since DELC was rare in infants [37] and Japanese male patients having DELC had shortened telomeres in peripheral white blood cells, again implicating aging [27]. Embryologic and vascular supply disorders are also suggested by the same genetically originated end-arterioles and similar leukocyte antigen subtypes for both DELC and CAD [33]. Some authors suggested that DELC might be the earliest manifestation of a generalized vascular disease [35] and subclinical atherosclerosis [36]. For the DELC was also associated with CAD surrogates as brachial-ankle pulse wave velocity and aortic intima-media thickness in subjects free of clinical cardiovascular disease. The molecular biology research demonstrated a common pathophysiologic relation between DELC and CAD, because earlobe collagen consists of peptide chains resembling those present on scavenger macrophages receptor used for the ingestion of atheromatous cholesterol [38].

We would like to state that with solely one ethnic population, relatively small sample size and single center design in this study, the result may not be generalized to out of patients and other population.

## Conclusions

To the best of our knowledge, this is the first report disclosed the significant association between DELC and CAD diagnosed by CAG in Chinese. Agreeing with most of the previous studies, this phenomenon may be conducive to the identification of the persons at risk in the earlier stages and adoption of active primary or secondary prevention for atherosclerosis.

## Competing interests

The authors declare that they have no competing interests.

## Authors’ contributions

XLW and DYY designed and interpreted all the results of the investigation. WHC, PL and MLJ carried out patients enrollment and data records. XLW and YSZ conducted the statistic analysis. The manuscript was initially drafted and written by XLW and DYY. All authors read and approved the final manuscript.

## Pre-publication history

The pre-publication history for this paper can be accessed here:

http://www.biomedcentral.com/1471-2261/14/43/prepub
